# Fluorescence guidance using near-infrared fluorescent clips in robotic rectal surgery: a case series

**DOI:** 10.1007/s00384-024-04615-w

**Published:** 2024-03-23

**Authors:** Satoshi Narihiro, Syunsuke Nakashima, Mutsumi Kazi, Tomotaka Kumamoto, Kazuo Kitagawa, Naoki Toya, Ken Eto

**Affiliations:** 1 Department of Surgery, The Jikei University Kashiwa Hospital, 163-1 Kashiwashita, Kashiwa, Chiba 277-8567 Japan; 2 Department of Surgery, The Jikei University School of Medicine, Minato-ku, Tokyo, Japan

**Keywords:** Rectal cancer, Fluorescence-guided methods, Robotic surgery, Near-infrared fluorescent clips

## Abstract

**Purpose:**

Tattoo markings are often used as preoperative markers for colorectal cancer. However, scattered ink markings adversely affect tumor site recognition intraoperatively; therefore, interventions for rectal cancer may lead to an inaccurate distal resection margin (DRM) and incomplete total mesorectal excision (TME). This is the first case series of fluorescence-guided robotic rectal surgery in which near-infrared fluorescence clips (NIRFCs) were used to localize rectal cancer lesions.

**Methods:**

We enrolled 20 consecutive patients who underwent robotic surgery for rectal cancer between December 2022 and December 2023 in the current study. The primary endpoints were the rate of intraoperative clip detection and its usefulness for marking the tumor site. Secondary endpoints were oncological assessments, including DRM and the number of lymph nodes.

**Results:**

Clip locations were confirmed in 17 of 20 (85%) patients. NIRFCs were not detected in 3 out of 7 patients who underwent preoperative chemoradiation therapy. No adverse events, including bleeding or perforation, were observed at the time of clipping, and no clips were lost. The median DRM was 55 mm (range, 22–86 mm) for rectosigmoid (Rs), 33 mm (range, 16–60 mm) for upper rectum (Ra), and 20 mm (range, 17–30 mm) for low rectum (Rb). The median number of lymph nodes was 13 (range, 10–21).

**Conclusion:**

The rate of intraoperative clip detection, oncological assessment, including DRM, and the number of lymph nodes indicate that the utility of fluorescence-guided methods with NIRFCs is feasible for rectal cancer.

**Supplementary Information:**

The online version contains supplementary material available at 10.1007/s00384-024-04615-w.

## Introduction

The accuracy of tumor marking is a critical issue in laparoscopic and robotic colorectal surgeries. Accuracy of tumor marking has been a prerequisite of successful outcomes in colorectal laparoscopic and robotic surgeries. Tumor marking is a well-recognized factor that can impact patients undergoing surgery. Inappropriate markings may reduce the quality of the surgery and may affect prognosis, including recurrence.

Radical surgical resection and preservation of anal function are the main objectives of rectal cancer surgery. Regarding the curability rate of the interventions, rectal cancer has a higher rate of local recurrence than colon cancer [1, 2]; hence, it is critical to improve surgical quality. Distal resection margins (DRMs) and total mesorectal excision (TME) have been proposed as metrics of surgical quality in rectal cancer. An optimal DRM size is essential to eliminate lymph node metastases in the mesentery [3, 4], and TME is the standard treatment for patients with low rectal cancer [5]. Considering anal function, low rectal cancer located near the anorectal junction was previously treated with abdominoperineal resection; however, anal preservation was not achieved. However, improvements in surgery, including intersphincteric resection, have enabled additional anus-preserving surgical procedures. However, various factors, including narrow pelvis, obesity, and preoperative therapy, reportedly contribute to the degree of difficulty in surgery. In addition, no consensus has been reached regarding preoperative marking methods to be used for rectal cancer.

Tattoo markings are often used as preoperative markers for colorectal cancer. However, tattoo markings carry the risk of accidental intestinal puncture, peritoneal scattering, or injury to other abdominal organs. Especially, the scattering of ink has been found to adversely impact tumor site recognition, which may lead to inaccurate DRMs and incomplete TMEs in patients with rectal cancer [6, 7]. Preoperative chemoradiation therapy (CRT) is a standard treatment option for patients with rectal cancer [5, 8, 9], which can reduce the size of primary lesions. Consequently, the intestinal resection length is shortened, preserving anal function. In such cases, tattooing is considered unsuitable as a marking method for rectal cancer.

Robotic surgery for rectal cancer is becoming increasingly popular. The Da Vinci^®^ Xi surgical system is an integrated fluorescence imaging (firefly technology) system, which comprises an endoscope camera with an infrared excitation laser (805 nm) that visualizes infrared light (830 nm) [10–12] and firefly technology to enable fluorescence-guided surgery using near-infrared fluorescence clips (NIRFCs; ZEOCLIP FS^®^, Zeon Medical, Tokyo, Japan) [7]. To achieve a complete TME and accurate DRM, tumor site marking with NIRFC may be utilized instead of tattoo marking, which results in ink scattering. We have previously reported the use of NIRFCs as a preoperative marking method [7]. NIRFCs allow accurate, real-time monitoring of lesion location and sufficient intestinal resection by grasping the lesion precisely. In addition, the use of NIRFCs facilitates the easy recognition and of TME layers.

Herein, we present the first case series of fluorescence-guided robotic rectal surgery utilizing firefly technology with NIRFCs to localize rectal cancer lesions. Additionally, we evaluated the feasibility and safety of fluorescence-guided robotic rectal surgeries.

## Materials and methods

**Fig. 1:**
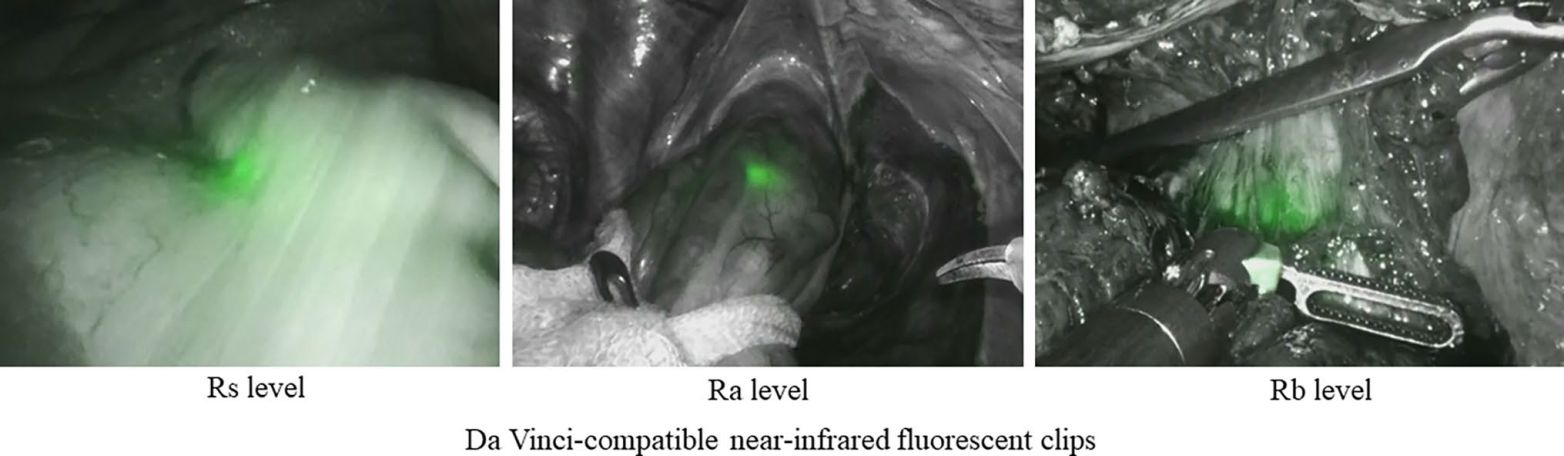
Locations of the Da Vinci–compatible near-infrared fluorescence clips (NIRFCs) confirmed using firefly technology in each area of the rectal cancer

**Fig. 2:**
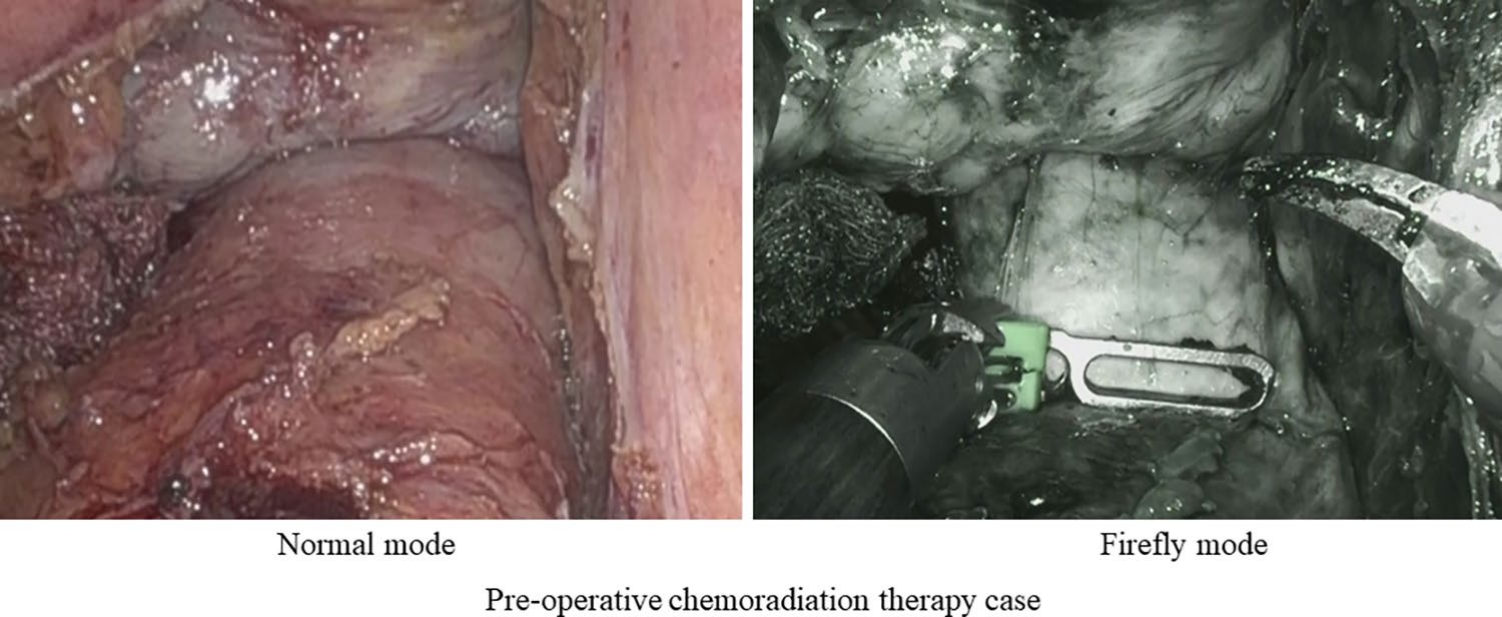
Rectal wall after preoperative chemoradiation therapy (CRT) shows thickening; thus, the tumor lesion could not be identified

**Fig. 3:**
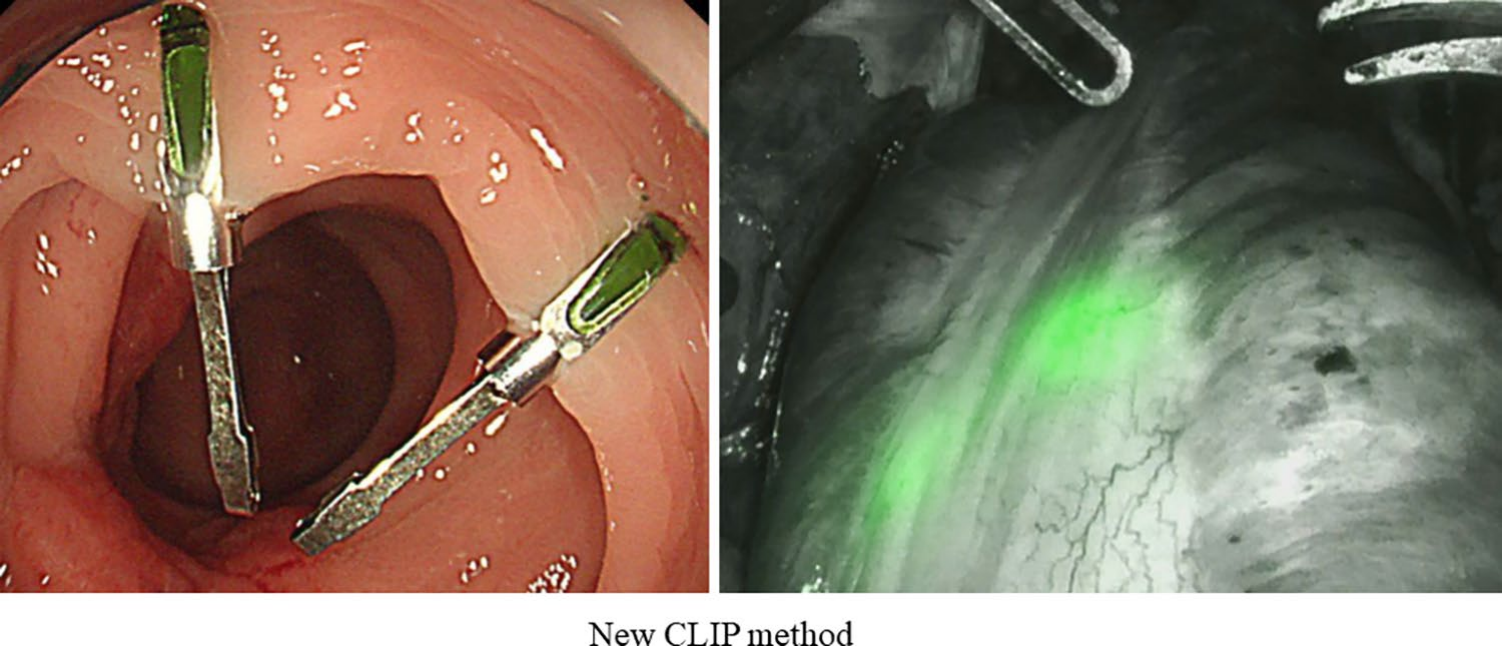
The proposed new clip attachment method improves visualization of near-infrared signals by concentrating the clip only on the ventral side

Twenty consecutive patients who underwent robotic surgery for rectal cancer between December 2022 and December 2023 were enrolled. No indication was excluded for patient selection. The study was approved by the Institutional Review Board (no. 30-249[9270]) prior to patient enrolment. Written informed consent was obtained from all patients for participation in the study. All research participants provided informed consent for the publication of the images in Figs. 1, 2, and 3. All patients were treated and followed up at the same medical academic institution. This was a prospective, single-center study. For all patients, clip placement was performed by the same endoscopist (SA), and robotic surgery was performed by two surgeons (SN and KK), i.e., senior colon and rectal surgery specialists, respectively.

For all patients, NIRFCs were placed during a colonoscopy performed one day before surgery. All patients underwent bowel preparation before colonoscopy. In each case, four clips were attached to the intestinal mucosa intraluminally, close to the distal extent of the lesion, four clips for each lesion, each clip at a 90° angle from the others. This method of clip detection is the same as that employed in our previous report [6, 7].

NIRFC is a newly designed tumor marking tool for robotic surgery, manufactured and approved for clinical use in 2019 (reg. no. 13B1X001111000020), with peak excitation and fluorescence wavelengths of 760 and 790 mms, respectively. NIRFC is fabricated from stainless steel, comprises polycarbonate, and is 12.5 mm in size. It has a fluorophore resin–filled tip, which emits near-infrared signals when excited. If placed within the lumen of the intestinal tract, these signals can be detected from the serosal side, thereby allowing clip localization. The primary endpoints of this study were the rate of intraoperative clip detection and its usefulness for preoperative marking of the tumor site. The secondary endpoints were oncological assessments, including DRM and the number of lymph nodes. This manuscript adheres to relevant PROCESS guidelines.

## Results

All 20 enrolled patients underwent robotic rectal surgery. There were 14 males and 6 females, with a median age of 70 years (range, 49–83 years). The median body mass index (BMI) was 23.5 kg/m^2^ (range, 16.7–31.2 kg/m^2^). The tumors were located at the rectosigmoid (Rs) (*n* = 9), upper rectum (Ra) (*n* = 7), and low rectum (Rb) (*n* = 4) levels (Fig. 1). With regard to cancer progression, five patients had stage I tumors, six had stage II tumors, six had stage III tumors, three had stage IV tumors, and seven (35%) underwent preoperative CRT (Table 1).

**Table 1:** Patient and surgical characteristics

	*n* = 20
Sex (M:F)	14:6
Median age (range), years	70 (49–83)
BMI (range) kg/m^2^	23.5 (16.7–31.2)
Comorbidity	7 (35%)
Open surgery history	3 (15%)
Tumor lesion (Rs: Ra: Rb)	9 (45%): 7 (35%): 4 (20%)
Clinical stage (I:II: III: IV)	5 (25%): 6 (30%): 6 (30%): 3 (15%)
T4	1
Preoperative CRT	7 (35%)
Thick fatty tissue deposits	1
Tumor size (mm)	34 (11–95)
Type of operation	
HAR	8 (40%)
LAR	11 (55%)
Hartmann’s	1 (5%)
Protective ileostomy	8 (40%)

Numbers reported as median (range) or *n* (%)

*BMI* body mass index, *CRT* chemoradiation therapy, *HAR* high anterior resection, *LAR* low anterior resection

All patients underwent a preoperative colonoscopy on the day preceding the surgery. Clip locations were confirmed using the firefly technology in 17 of 20 (85%) patients. The clip location in the Rs area was confirmed in nine patients (100%). The Ra area was confirmed in 4 patients (100%), and the Ra area with preoperative CRT was confirmed in 1 patient (33%). The Rb area was confirmed in 1 patient (100%), and the Rb area with preoperative CRT was confirmed in 2 patients (67%). One patient had T4 tumor progression, while another had thick fatty tissue deposits around the colon; however, these potentially interfering issues did not affect NIRFC detection. However, 7 (35%) patients underwent preoperative CRT, and NIRFC detection was affected in 3 of these patients (Table 2). We did not observe any cases in which the clips were dislodged or caught in the linear stapler at the time of intestinal dissection. No adverse events, including bleeding or perforation, were observed at the time of clip insertion, and no clips slipped off. The median DRM was 55 mm (range, 22–86 mm) for Rs, 33 mm (range, 16–60 mm) for Ra, and 20 mm (range, 17–30 mm) for Rb. The median number of lymph nodes was 13 (range, 10–21) (Table 3).

**Table 2:** Clip recognition rate

		*n* = 20
	Positive	Negative
Total	17 (85%)	3 (15%)
Rs	9 (100%)	0
Ra	4 (100%)	0
Ra + Preoperative CRT	1 (33%)	2 (67%)
Rb	1 (100%)	0
Rb + Preoperative CRT	2 (67%)	1 (33%)

Numbers reported as *n* (%)

*CRT* chemoradiation therapy, *Ra* upper rectum, *Rb* lower rectum, *Rs* rectosigmoid

**Table 3:** Peri-operative and postoperative outcomes

Outcome	(*n* = 20)
Operation time (min)	378.5 (257–612)
Blood loss (mL)	55 (5–200)
Intraoperative complications	0
Anastomotic leakage	0
Reoperation	0
Mortality	0
Distal resection margin (mm)	
Rs	55 (22–86)
Ra	33 (16–60)
Rb	20 (17–30)
Number of harvested lymph nodes	13 (10–21)
Length of postoperative hospital stay (days)	14.5 (9–24)

Numbers reported as median (range)

*Ra* upper rectum, *Rb* lower rectum, *Rs* rectosigmoid

## Discussion

In the current study, we demonstrated that clip locations could be confirmed using the firefly technology in 17 of 20 (85%) patients. In addition, there were no adverse events due to clip attachment or clips dropping out. In the oncological assessment, the ideal size of the DRM was sufficient, and the number of lymph nodes removed during dissection was precise. These results indicate that fluorescence-guided methods using NIRFCs are safe and feasible for treating rectal cancer.

The application of tattoo markings for preoperative marking has been associated with various disadvantages, such as unclear range, excessively thin marking, and ink scattering. In rectal cancer, a shorter DRM and an unsuitable TME increase the risk of local recurrence and decrease the overall survival rate [3–5]. An unclear range or excessively thin markings make it difficult to recognize the optimal DRM. Ink scattering complicates the identification of the optimal DRM and TME layers. Therefore, it is unsuitable for the treatment of rectal cancer. To achieve a complete TME and optimal DRM, tumor site marking with NIRFCs may be used as an alternative to tattoo marking. As a notable advantage, the use of NIFRCs as a preoperative marking method facilitates precise tumor extraction [6, 7, 13]. Therefore, we attempted to apply NIFRCs as a marking method for rectal cancer. This has oncological benefits in rectal cancer, allowing the selection of the optimal intestinal incision length (optimal DRM) and correct dissection surface (correct TME layer). To demonstrate the benefits of NIFRCs in rectal cancer, it is important to carefully evaluate their visibility. Although one patient with T4 tumor progression and one patient with thick fatty tissue deposits around the rectum presented issues for potential interference, these did not affect the detection of NIFRCs.

Despite an almost complete retention rate among all patients, tumor locations could not be identified in three patients, and preoperative CRT was performed in all three cases. CRT is known to induce inflammation, necrosis, and fibrosis, resulting in thickening of the rectal wall [14, 15] (Fig. 2). Currently, the use of NIFRCs is not recommended for patients who have undergone CRT. In patients who underwent CRT, it was necessary to obtain the strongest near-infrared signal, and three approaches have been employed. The first is to compress the intestinal tract to minimize soft tissue penetration. The second approach is to position the laparoscope vertically to obtain a sufficient angle of fluorescence excitation to detect the clip; the intestine must be mobilized before proper laparoscope positioning. Third, we considered a new clip-attachment method, which made it easier to obtain near-infrared signals by concentrating the clip on the ventral side only rather than hitting the entire circumference (Fig. 3).

This study had several limitations. First, owing to the small sample size and prospective, single-center, non-randomized design, potential bias could not be eliminated. Second, all operative procedures were performed by two surgeons and endoscopists; such a setting might have been a possible source of optimism bias. Third, we could not evaluate circumferential resection margins (CRMs) for oncological assessment. Similar to DRM and TME, the CRM influences the local recurrence rate. After preoperative treatment, the 5-year local recurrence rate for a CRM measuring > 1 mm was substantially lower than for a CRM ≤ 1 mm [16, 17]; hence, we need to evaluate the CRM as an oncological endpoint in the future. Fourth, NIFRCs are expensive (the required cost is approximately $100 per 1 fluorescent clip); therefore, we need to reduce the number of clip placements as feasible. Nevertheless, the advantages of NIFRCs need to be highlighted, including optimal intestinal incision length (optimal DRM) and correct dissection surface (correct TME layer). Accordingly, it is important to analyze the oncological outcomes and total costs of both procedures.

In conclusion, the fluorescence-guided method using NIRFCs was safe and feasible for rectal cancer; hence, we believe that this method using NIRFCs can be a promising surgical option in rectal cancer resection.

## Supplementary Information

Below is the link to the electronic supplementary material.

Supplementary file1 (DOCX 33.1 KB)

## Data Availability

No datasets were generated or analysed during the current study.
